# Low Back Pain Among Health Sciences Undergraduates: Results Obtained from a Machine-Learning Analysis

**DOI:** 10.3390/jcm14062046

**Published:** 2025-03-17

**Authors:** Janan Abbas, Malik Yousef, Kamal Hamoud, Katherin Joubran

**Affiliations:** 1 Department of Physical Therapy, Zefat Academic College, Zefat 13206, Israel; kamalh@zefat.ac.il (K.H.); katherin.jo@zefat.ac.il (K.J.); 2 Department of Information Systems, Zefat Academic College, Zefat 13206, Israel; malik.yousef@zefat.ac.il

**Keywords:** health science students, low back pain, machine learning, history of pain, physical activity

## Abstract

**Background and objective.** Low back pain (LBP) is considered the most common and challenging disorder in health care. Although its incidence increases with age, a student’s sedentary behavior could contribute to this risk. Through machine learning (ML), advanced algorithms can analyze complex patterns in health data, enabling accurate prediction and targeted prevention of medical conditions such as LBP. This study aims to detect the factors associated with LBP among health sciences students. **Methods.** A self-administered modified version of the Standardized Nordic Questionnaire was completed by 222 freshman health sciences students from May to June 2022. A supervised random forest algorithm was utilized to analyze data and prioritize the importance of variables related to LBP. The model’s predictive capability was further visualized using a decision tree to identify high-risk patterns and associations. **Results.** A total of 197/222 (88.7%) students participated in this study, most of whom (75%) were female. Their mean age and body mass index were 23 ± 3.8 and 23 ± 3.5, respectively. In this group, 46% (n = 90) of the students reported having experienced LBP in the last month, 15% (n = 30) were smokers, and 60% (n = 119) were involved in prolonged sitting (more than 3 h per day). The decision tree of ML revealed that a history of pain (score = 1), as well as disability (score= 0.34) and physical activity (score = 0.21), were significantly associated with LBP. **Conclusions.** Approximately 46% of the health science students reported LBP in the last month, and a machine-learning approach highlighted a history of pain as the most significant factor related to LBP.

## 1. Introduction

Low back pain (LBP) is commonly recognized as a health, social, and economic burden in Western countries. The condition covers different types of pain and involves a spectrum of structures (e.g., muscles and discs). It has been reported that mechanical LBP, which is considered the most common type, could lead to pain chronicity [1]. It is also accepted that LBP increases with age [1,2], and its prevalence according to the National Center of Health Statistics at younger (18–44 years) and older ages (45–65 years) is 23% and 35%, respectively [2]. It is worth noting that LBP is a multifactorial component that includes biological, environmental, and psychological factors [3]. Growing evidence suggests that student lifestyles, particularly those in health science programs, lead to a higher prevalence of LBP than their counterpart in the general population [4,5]. For example, the 12-month prevalence of LBP among nursing students was found to be high (65%), whereas the reported rate among student athletes was 39% [6]. Furthermore, some studies reported that being a physiotherapy student was considered to be a risk factor for LBP [7,8]. These findings could be associated with the fact that health science students possess highly demanding curricula (e.g., loss of sleep hours), poor working posture, manual handling activities during training, and stressful experiences [8,9,10]. However, data regarding the specific variables that greatly stimulate LBP among health science students are still unclear [10,11,12,13]. For example, two prospective cohort studies [10,11] among nursing students found diverse predictive variables for LBP.

Machine learning (ML) is continually gaining importance in medical predictive analytics and has numerous advantages compared to conventional analyses such as logistic regression [14]. ML algorithms can expose highly complex interactions among variables that allow for more accurate medical prognosis, as well as other novel applications [15,16]. ML can be divided mainly into supervised and unsupervised models. A common supervised model is that of decision tree (DT) learning, whose classification and regression implement a grouping or a regression task, which is more visible and easier to understand than other modalities [17]. Additionally, DT can learn highly irregular patterns, with low bias and high variance for the training set, boosting the model’s performance [18]. Most studies on ML in spinal disorders are in the domain of diagnosis, followed by prognosis, prediction, and biomechanics for spinal applications [19]. A recent systematic review showed that ML has been used only for the recovery prediction and treatment of LBP [20]. The authors concluded that ML could also enhance the ability to detect patterns of clinical characteristics in LBP [20] even though little existing ML research predicts its onset. Understanding the risk factors of LBP among undergraduates of health science, who are more likely to engage in unhealthy behaviors that adversely affect their well-being, can help in implementing suitable preventive or treatment measures to attenuate LBP chronicity in future daily function and practice.

Thus, this study aims to investigate the factors contributing to LBP among health science students using the ML approach. By applying a random forest algorithm, the study seeks to uncover complex variable interactions, prioritize risk factors, and provide actionable insights for preventive strategies tailored to this population.

## 2. Materials and Methods

Study design. A cross-sectional study of 222 freshman undergraduates in health sciences (nursing, physical therapy, medical laboratory science, and emergency medical services) from the Zefat Academic College in the north of Israel who were enrolled between May and June 2022. There were no exclusion criteria other than the inability to fill out the hardcopy self-reported questionnaire. We considered only the freshman class, as we plan to follow the participants throughout their academic years. The participants were recruited in their classrooms by one of the study’s assessors at the end of that course in the presence of their instructor. A consent form, which included the purpose of the study and the right of the student to withdraw at any time, was received from each participant.

The study was conducted in adherence to the Helsinki Declaration, and it was approved by the Departmental Research Ethics Committee at Zefat Academic College (no. 19-2022).

### 2.1. Instruments and Measures

A structured and anonymous questionnaire according to the modified validated Standardised Nordic Questionnaire [21] that seeks information on sociodemographic characteristics and factors such as sedentary behavior and smoking habits was used [22] (Table 1). Physical activities were also recorded and were adapted from the American College of Sports Medicine guidelines [23]. These were graded from 0 = no physical activity to 5 = high frequency of physical activity. The students were asked if they had ever suffered LBP at some point in their life (lifetime prevalence) and if they had ever been hospitalized due to LBP. Those who had experienced LBP in the last year (based on the response “yes” to the question) were asked specific questions about pain frequency (graded from 1 = daily to 4 = rarely/never) and medication consumption, as well as whether they were seeking care and disability according to the Oswestry Disability Index (ODI) [24].

In addition, the participants were asked about variable-related stress [21] in the last month (e.g., study and personal issues), and were divided into two categories based on their response to the question: “What is your daily stress level?” (a = *very high to quite high*, b = *low to none*). Their LBP in the last month was also recorded, and this was considered positive if it had lasted at least 12 h and its intensity scale according to the numeric rating [25] was above five.

### 2.2. Statistical and Supervised Machine-Learning Analyses

In analyzing our data, we first used statistical methods with IBM SPSS version 25. We checked whether the numerical data followed a normal distribution and conducted descriptive analyses for both numerical and categorical variables. Next, we applied machine learning (ML) to identify key factors that influence LBP. Specifically, we used the Random Forest (RF) algorithm, a powerful ML method for complex data analysis [26]. RF comprises many DTs, which are simple models that mimic human decision-making [27]. Each DT asks a series of yes/no questions to classify data. For example, a tree might first ask a person if they sit for long hours and then ask their age before predicting their risk for LBP. RF combines multiple DTs to improve accuracy and find the most critical factors related to LBP. Potential variables such as age, activity level, and sedentary behavior were identified from previous studies. To ensure our results were reliable and generalizable, we implemented a rigorous validation approach using 100-fold Monte Carlo cross-validation [28]. We randomly split the dataset into training (70%) and testing (30%) sets for each fold, maintaining the class distribution across splits. This technique helps evaluate a model’s stability by repeatedly training and testing it on different subsets of the data, minimizing potential bias from a single arbitrary split.

We evaluated how well the model performed using several measures: sensitivity—how well it identified people with LBP, specificity—how well it identified those without LBP, precision—how accurate the positive predictions were, accuracy—the overall correctness of the predictions, and F-measure—the balance between precision and sensitivity. The area under the receiver operating characteristics curve (AUROC) was also considered for the test dataset to be a strong indicator of performance for classifiers in imbalanced datasets.

A total of 197 students participated in this study. For the DT learning process, we divided the participants into two groups: 90 with LBP and 107 without LBP. The optimal feature was selected recursively, and the training data were segmented according to this feature to ensure the best classification process. This process corresponds to the division of the feature space and the construction of the DT.

First, we constructed the root node, where all training data were placed. An optimal feature was chosen, and the training dataset was divided into subsets according to this feature, ensuring the best classification under the given conditions. If these subsets were correctly classified, they were assigned to leaf nodes. If any subset remained unclassified, a new optimal feature was selected, and the process continued until all data were classified or no more suitable features were available. Finally, each subset was assigned to the leaf nodes, completing the DT structure [29]. The KNIME platform was used with default parameters, which was deemed sufficient after initial testing showed stable performance.

## 3. Results

### 3.1. Student Characteristics

**Figure 1:**
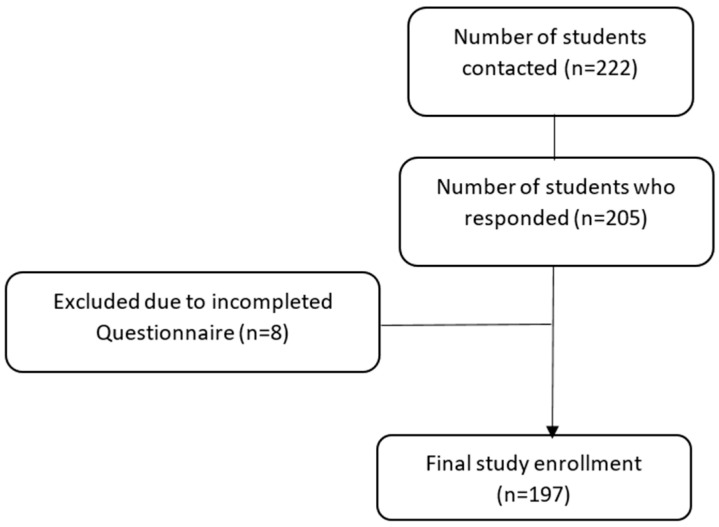
Flow chart for the study sample.

One hundred ninety-seven students (88.7%) responded and completed the questionnaire (Figure 1). The demographic and sedentary features of the participants are presented in Table 1. The mean age and body mass index (BMI) values for all the participants were 23 ± 3.8 and 23 ± 3.5, respectively. Seventy-five percent were females (n = 148) and 45% (n = 89) were from the nursing department. The majority (93%) of participants were single, and 15% were habitual smokers. Additionally, 73% defined themselves as secular and traditional, 60% were involved in prolonged daily sitting (more than 3 h), and most of them (85%, n = 167) reported that they experienced study-related stress.

In our series, 55% (n = 108) of the students took part in sports activities with diverse frequencies: 7% (n = 14) with low frequency (at least 20 min to 1 h weekly) and 19% (n = 37) with high practice frequency (more than 3 h).

### 3.2. Low Back Pain

A high prevalence of students (74%, n = 146) have experienced LBP at some point in their lives, and 4% (n = 8) have been hospitalized because of such pain. In addition, 46% (n = 90) reported that they had suffered lower back pain in the last month (Table 2). The features of LBP during the last year were dispersed. For example, the frequency of LBP was as follows: 17% (n = 34) suffered almost daily, 24% (n = 47) once a week, 22% (n = 43) once a month, and 37% (n = 73) rarely or never. It was also found that 33% of the students (n = 65) reported disability, and about 20% (n = 39) were referred to a physician and/or used medication.

The assessment of the model performance such as accuracy and recall are presented in Table 3.

**Figure 2:**
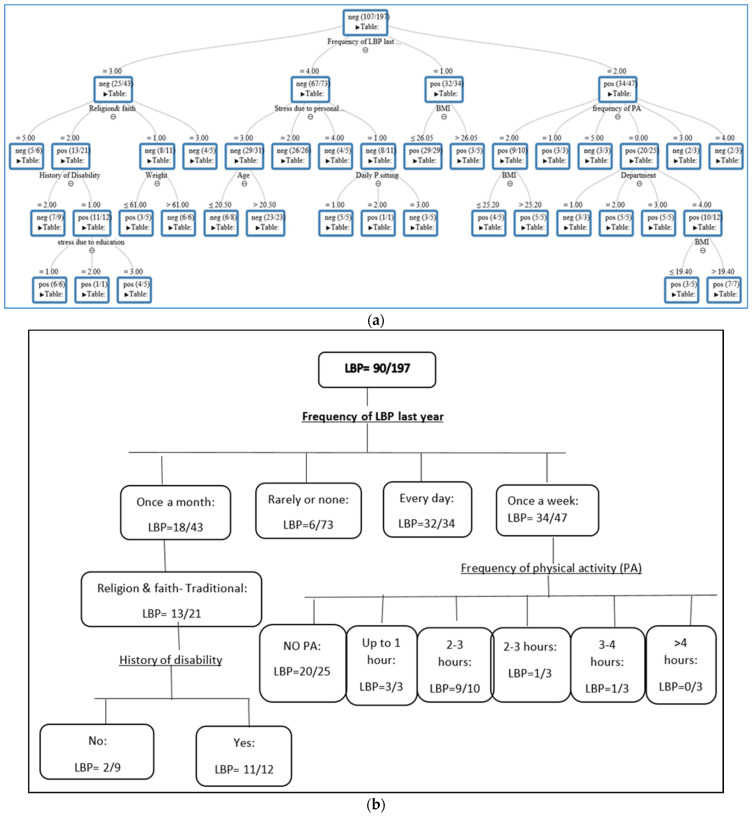
(**a**): Decision tree output of the random forest for predicting 1-month LBP obtained by the given variables. Neg = negative, pos = positive. PA—physical activity; (**b**): the main variables significantly associated with LBP according to the decision tree outcome.

The DT represents the variables that are associated with LBP. As depicted in Figure 2a, the tree comprises internal nodes (conditions), branches (decisions), and leaves (outcomes). In general, variables that appear at the top of the tree (e.g., frequency of LBP) have a greater impact on LBP than those in the bottom nodes, such as department or BMI. To understand the DT output in Figure 2a, we illustrated its main findings in Figure 2b. We found that 90 out of the 197 students reported LBP in the last month. We also noticed that the “frequency of LBP in the last year” was considered to be at the “peak” of the tree, having the greatest impact on LBP (score = 1, Table 4). Additionally, among those who suffered from LBP more regularly (daily), 32 students out of 34 had LBP (94%), and when the frequency was reported as “rarely”, this percentage decreased to 8% (6 out of 73). This result implies that once the person begins to experience LBP more often, the likelihood of its recurrence increases. We also observed that a history of “disability” and physical activity (PA) influenced the onset of LBP, with scores of 0.34 and 0.21, respectively (Table 4). For example, 11 students out of 12 reported LBP if they met the following conditions: (a) had pain frequency at least “once a month”, (b) were traditionally religious, and (c) had experienced disability. Notably, the contribution of variables appearing in the later example’s internal nodes, such as religion (traditional), is partial since only 13 students out of 21 reported LBP (Figure 2b) (score = 0.13, Table 4). In addition, 20 students out of 25 suffered from LBP if their pain frequency was “once a week” and they did not engage in PA. The scores of each variable associated with LBP in the DT are depicted in Table 4.

## 4. Discussion

This cross-sectional study aimed to verify the associated variables for LBP among freshman health sciences students using machine learning. Contrary to previous studies that applied logistic regression analysis with well-known limitations (e.g., unstable variable selection), here, we used a random forest of ML algorithms. To the best of our knowledge, few studies have addressed the risk factors for LBP using this approach [30,31,32].

Although the utilization of ML for detecting LBP is still in its infancy, it was recently reported that supervised ML compared with other analyses (e.g., regression) could predict LBP among college students from a questionnaire with high accuracy [31]. Shim and colleagues (2021) also noted that ML could effectively identify populations at high risk of chronic LBP [32].

This study showed lifetime and 1-month prevalences of LBP of 74% and 46%, respectively. This finding falls within the range (31% to 84%) previously reported among health sciences undergraduates worldwide [7,13,33,34,35,36,37,38,39]. The lifetime prevalence among these undergraduates, such as those studying nursing and physiotherapy, was about 50% [7,13]. It was reported that about 84% of a freshman class in health sciences (n = 153) suffered from LBP [36]. Nyland and Grimmer also reported a 31% 1-month prevalence among first-class physiotherapy students [7].

Our results confirm that a history of pain (frequency) has the greatest relationship (score = 1) with LBP. However, while this finding is in agreement with some studies [10,40,41], it contradicts others [11,13,34,35,42,43,44]. A systematic review study in the general population showed that a history of previous episodes of LBP was the only predicting factor for the recurrence of LBP [40]. A prospective cohort study (over 4 years) among nursing students also reported that a history of LBP was considered a prediction factor for new episodes [10]. In contrast, other studies revealed that smoking, advanced physical activity, higher stress levels, a sedentary lifestyle, and mal-posture were independent factors for LBP among nursing students [11,35,43]. Notably, factors associated with such back pain are inconsistent due to high-risk biases, such as heterogeneity of the methodology and sample features (e.g., students’ department, general population) and/or a small study sample.

This study also found that disability in the last year and a lack of physical activity can predict LBP. This association between a disability history and LBP was previously confirmed [44,45]. For example, in a prospective cohort study in a working population with a 3-year follow-up period, it was found that high disability due to LBP was a prognostic factor for its recurrence [44]. As mentioned, data regarding physical activity and LBP are still conflicting [12,46,47,48]. For example, Felemban et al. [48] and Amelot et al. [12] found that exercise and walking at least 30 min a day significantly improved LBP among a group of dental and medical students. Conversely, an Australian cross-sectional study among nursing students revealed that vigorous physical activity contributed to LBP [46]. It is widely recognized that physical activity has numerous health benefits related to physiological and psychological health [49,50,51]. Moreover, it is well-accepted that physical activity can reduce the risk factors of various chronic diseases such as those that are cardiovascular and musculoskeletal, as well as diabetes [50,52]. Thus, the impact of physical activity on LBP may depend on the activity type and the degree of loading on the lumbar spine [53,54]. One related review [55] suggested that the type and intensity of physical activity should be considered when measuring the association with LBP. A recent systematic and meta-analysis review reported that a medium activity level was associated with a lower prevalence of LBP [56], and the authors recommended moderate doses of physical activity when managing and preventing LBP.

The fact that 55% of our participants were involved in physical activity is in line with a review from Keating and colleagues that 40–50% of college students are physically inactive [57]. In addition, sufficient evidence has been found to indicate that most university students have an unhealthy lifestyle, with poor levels of physical activity (PA) [58,59]. These findings are not surprising and highlight what has previously been reported regarding the need for PA intervention programs for these students [60,61].

## 5. Limitations of the Study

Despite the widespread use and good validation of the Standardized Nordic Questionnaire (SNQ), it has some notable disadvantages. While it effectively screens for musculoskeletal symptoms such as LBP, it lacks diagnostic specificity and cannot differentiate between various pathologies causing similar pain patterns [62]. The SNQ relies entirely on self-reporting, introducing potential recall bias and subjective interpretation of symptoms, especially when respondents report pain over the previous 12-month period [63]. Its simplistic pain assessment fails to capture the multidimensional nature of pain, missing important aspects like pain duration, patterns, and psychological factors that influence pain perception. Cultural and linguistic aspects as well as socioeconomic factors can significantly affect how pain is reported and understood across different populations, potentially compromising cross-cultural validity despite translations. Additionally, the SNQ lacks psychometric refinement compared to newer instruments, noting that while broadly applicable, it has less robust reliability and validity metrics than more recently developed specialized assessment tools [64]. The SNQ also overlooks important contextual factors like work environment, biomechanical exposures, and psychological considerations that contribute to musculoskeletal disorders. It was also pointed out that the questionnaire provides insufficient information for clinical decision-making or treatment planning without supplementary assessments [65]. Finally, the instrument may not reflect current musculoskeletal disorders or contemporary occupational health concerns.

Notably, as the participants lack diversity (e.g., health science undergraduates, environmental), this may cause the model to miss relevant predictors in underrepresented groups [66]. The fact that the selected variables were based on previous studies could be misleading to favor variables aligned with existing clinical beliefs about LBP [67]. Additionally, causal relationships are challenging in cross-sectional studies, where decision trees might misidentify the consequences of LBP as predictors rather than actual risk factors [68]. Additionally, the study was conducted at a single academic institution in the north of the State of Israel, which limits its generalizability. As LBP is a multifactorial phenomenon, some related factors such as spinal deformity, depression, socioeconomic background, and medical history were not addressed in this study.

We believe that our findings underscore the potential of ML to predict and prioritize LBP risk factors, enabling targeted interventions. For instance, academic institutions could design preventive measures, such as tailored PA programs or therapeutic exercise protocols including automobilization [69] for high-risk students identified by the model. One can also assume that stakeholders should dedicate “physical activity time” to the student curricula to reduce the risk and chronicity of future LBP. We anticipate that further studies are needed using ML methods in different academic centers and cultures to check for repeatable results and to establish basic predictive factors for LBP among undergraduates.

## 6. Conclusions

The current study found that the 1-month prevalence of LBP among freshman health sciences undergraduates was 46%. The random forest algorithm indicates that a history of pain frequency has the greatest impact on LBP. In addition, physical activity and a history of disability were also associated with LBP.

## Figures and Tables

**Table 1:** Sample size characteristics.

% (n)/or Mean ± SD	
89 (197)	Response rate
25 (49)	Male
75 (148)	Female
	Department:
45 (89)	Nursing
21 (41)	Physical Therapy
19 (37)	Medical Lab
15(30)	Emergency Medical Services
23 ± 3.8	Mean age (year)
23 ± 3.5	Mean BMI (kg/m^2^)
91 (179)	Dominant right hand
	Marital status:
93 (184)	Single
7 (13)	Other
15 (30)	Smoking
	Religion & faith:
30 (60)	Secular
43 (84)	Traditional
13 (26)	Religious/orthodox
14 (27)	Other
55 (108)	Those with physical activity (wk):
7 (14)	20 min to 1 h
19 (37)	1–2 h
10 (20)	2–3 h
9 (18)	3–4 h
10 (19)	>4 h
	Prolonged daily sitting:
40 (78)	Up to 3 h
23 (45)	3–5 h
37 (74)	5 h>
	Total daily sitting:
32 (63)	Up to 6 h
30 (60)	6–8 h
38 (74)	>8 h
	* Study-related stress:
85 (167)	Very high–quite high
15 (30)	Little–none

* We present only study-related stress as this variable was most common compared to other types of stress.

**Table 2:** Low back pain (LBP) characteristics for the study group.

N (%)	
146 (74)	Those who experienced LBP at some point in their life
8 (4)	Those who had been hospitalized due to LBP
	LBP in the last year:
	1. Frequency of LBP:
34 (17)	Every day
47 (24)	Once a week
43 (22)	Once a month
73 (37)	Rarely/never
65 (33)	2. Disability
39 (20)	3. Seeking care
39 (20)	4. Medication consumption
90 (46)	1-month LBP

**Table 3:** Means and standard deviation (SD) of the model performance.

	Accuracy	Recall	Specificity	Precision	F1	Area Under Curve
Mean	0.84	0.93	0.76	0.79	0.84	0.88
SD	0.09	0.09	0.18	0.13	0.07	0.07

**Table 4:** Scores of the significant variables associated with LBP by the decision tree.

Variable	Score
Frequency of LBP	1
History of Disability	0.34
Frequency of physical activity (PA)	0.21
Weight	0.18
Religion and faith	0.13
Department	0.12

## Data Availability

The data presented in this study are available upon request from the corresponding author due to privacy reasons.
